# Special Issue on “Topological photonics and beyond: novel concepts and recent advances”

**DOI:** 10.1038/s41377-020-00437-x

**Published:** 2020-12-22

**Authors:** Zhigang Chen, Hrvoje Buljan, Daniel Leykam

**Affiliations:** 1 TEDA Applied Physics Institute and School of Physics, Nankai University, Tianjin, 300457 China; 2 Department of Physics and Astronomy, San Francisco State University, San Francisco, CA 94132 USA; 3 Department of Physics, Faculty of Science, University of Zagreb, Bijenicka cesta 32, 10000 Zagreb, Croatia; 4 Centre for Quantum Technologies, National University of Singapore, 3 Science Drive 2, Singapore, 117543 Singapore

**Keywords:** Other photonics, Electronics, photonics and device physics

The discovery of topological phases and topological insulators has revolutionized several fields of natural science, including condensed matter physics, materials science, and photonics. Topological concepts have been implemented in a variety of materials and in a broad range of wave systems ranging from electronic, atomic, photonic, plasmonic, polaritonic, to microwave, acoustic, and mechanical waves. About a dozen years ago, the concept of quantum Hall effect and topological insulators was introduced to the realm of photonics^1^, and the idea was soon demonstrated in a gyromagnetic photonic crystal^2^. Several years later, photonic topological insulators manifested as topologically protected edge states were realized in several pioneering experiments performed using different photonic systems^3–8^. Much of the topological feature of Bloch bands is revealed in momentum space, using the concepts such as the *Chern* number and *Berry* phase (as illustrated in the cover figure of this feature collection). By now, there have been numerous theoretical and experimental efforts in the study of nontrivial topological photonic systems, from fundamentals to applications, turning it into an ever-burgeoning research field of topological photonics^9–11^. Indeed, research activities on topological photonics have grown tremendously in the past decade, with numerous progress made in implementing topological phases of light using different platforms such as metamaterials, surface plasmons, exciton-polaritons, photonic crystals, waveguide lattices, and coupled cavities^9–14^. New findings and discoveries emerge rapidly in topologically protected edge states and corner states, topological phases in synthetic dimensions, high-dimensional topological insulators, nonlinear effects in topological systems, non-Hermitian topological photonics and quantum phenomena, and particularly, in the topological insulator lasers^15–24^.

During the summer of 2019, two international workshops on topological photonics and related topics were held severally in Tianjin, China and Daejeon, Korea, where the latest progress and emerging trends in this field were reported and discussed. It was a pleasant coincidence that the international workshop on “Topological Photonics and Beyond” was held at Nankai University—the alma mater of Shiing-Shen *Chern* and was filled by lectures from leading experts in the area around world including Michael *Berry*, as well as posters presented by many students and postdoc researchers. This Special Issue brings out a collection of some of the cutting-edge original research and review papers from these two workshops, including but not limited to higher-order and high-dimensional topological insulators, topological insulators in synthetic dimensions, photonic gauge fields and associated topological phenomena, nonlinear topological phenomena, and topological photonic applications such as topological valley-Hall lasers and Dirac-vortex fibers. In what follows, we briefly introduce each topic and highlight the key messages from the collected articles.

One of the most important advances in topological band theory in the last few years has been the discovery of higher-order topological phases. The distinguishing feature of higher-order topological phases is the existence of protected states on lower-dimensional boundaries of the system. Regular (1st-order) d-dimensional topological phases host protected modes on their (d-1)-dimensional boundaries, e.g., 2D systems hosting protected modes along their 1D edges. On the other hand, higher-order topological phases manifest protected modes on boundaries with an even lower dimension (d-2), (d-3), and so on^11,12^. A typical example is the existence of (0D) topological corner states in 2D systems. In this aspect, photonics is playing a leading role in the studies of higher-order topological phases, with many of the first experimental observations realized using photonic crystals or lattices with electromagnetic waves ranging from microwave to the optical frequency regime. In this Special Issue, Junsuk Rho and colleagues provided an overview of recent progress in this direction, focusing on 2D and 3D topological photonics, including higher-order topological phases^25^. In another article devoted to higher-order topological phases, Yuanjiang Xiang, Tie Jun Cui, Shuang Zhang, and colleagues demonstrated a 3D octupole topological phase hosting protected corner modes using a specially-designed electronic circuit^26^. The observed 0D corner state is topologically protected by anticommuting spatial symmetries of the circuit lattice, which could provide an effective platform for investigating other higher-order topological effects.

Another important direction in topological physics is the study of gapless topological phases, which are associated with protected band-crossing degeneracies in bulk band structures. Early examples of such “topological semimetals” include Weyl points of 3D band structures, and 2D Dirac points protected by sublattice symmetries in graphene-like honeycomb lattices. Other types of symmetries give rise to more exotic forms of protected band crossings such as nodal loops and knots^11^. In this Special Issue, Zhengyou Liu, Shuqi Chen, and colleagues reported the realization of phononic crystals for sound exhibiting 3D Dirac point degeneracies protected by a non-symmorphic crystal symmetry^27^, as well as the emergence of pairs of Weyl points upon breaking the protecting symmetry which leads to the surface states and helical interface states with features inherited by the resulting Weyl crystal^28^. In another paper, Che Ting Chan, Yuntian Chen, and colleagues predicted that topological photonic crystal systems can host novel classes of higher-order degeneracies protected by crystalline symmetries unique to electromagnetic waves and inaccessible using electronic condensed matter, including a new kind of symmetry-enforced degenerate points (spin-1 Dirac-like cones) as the nexuses of nodal lines and loops^29^.

Photonic systems have provided ideal platforms for studying the higher-dimensional topological phenomena discussed above, because photonic systems are not limited to their underlying spatial dimension. Another growing direction of research is the concept of synthetic dimensions, which emulates the physical dimensions using internal degrees of freedom such as the orbital angular momentum, cavity mode, and spatial or frequency mode of light. In this Special Issue, Shanhui Fan and colleagues proposed photonic higher-order topological insulators based on synthetic frequency dimensions emerging in temporally-modulated ring resonators, which leads to a flexible platform for implementing higher-order topological phases including the fourth-order topological phases inaccessible in systems with just real spatial dimensions^30^. In a related work, Andrey Sukhorukov and colleagues experimentally demonstrated how nonlinearity can be used to implement tunable couplings in synthetic frequency dimensions, including long-range couplings and synthetic gauge fields generating effective magnetic fluxes^31^. Such flexible synthetic topological platforms are useful and advantageous for exploring fundamental phenomena of high-dimensional topological physics and may find applications also in quantum communication and information processing.

Artificial gauge fields and associated topological phenomena have attracted increasing interest in the past decade, realized in many physical settings including photonics, cold atoms, and acoustic waves. Artificial gauge fields are particularly of interest, as they are analogous to the gauge (vector) potentials for electrons that led to the celebrated Aharonov–Bohm effect and many other phenomena. In this Special Issue, Mordechai Segev and colleagues introduced the generalized law of refraction and reflection at interfaces between different photonic artificial gauge fields^32^. The law of reflection and refraction of light at the interface of two different media, also known as the Snell’s law, is one of the first things one learns in a beginner’s optics book, but a fundamental problem not addressed before is how the Snell’s law would change at the interface of materials with artificial gauge fields. In this novel context, the authors used the symmetries in the system to obtain the generalized law kinematically and dynamically and demonstrated the concept in experiments.

In two articles of this Special Issue, experimental implementations of artificial gauge fields were realized with a honeycomb polariton lattice^33^ as well as a lattice of microwave resonators^34^. Specifically, Alberto Amo, Omar Jamadi, and colleagues reported the realization of a synthetic magnetic field for photons and polaritons in a strained honeycomb lattice of coupled semiconductor micropillars, which leads to formation of Landau levels at the Dirac points and related edge states^33^. Landau levels and unidirectional propagating edge states are a hallmark of the well-celebrated quantum Hall effect (QHE). The authors demonstrated analogous phenomena with photons and polaritons by engineering a uniaxial hopping gradient in the lattice thereby creating a strong synthetic magnetic field. Independently, Henning Schomerus, Matthieu Bellec, Fabrice Mortessagne, and colleagues used the concept of strained graphene in an array of coupled microwave resonators to create a tunable synthetic magnetic field, which leads to the formation of Landau level states analogous to those in the QHE in graphene lattices, and the flat-band (pseudo-) Landau levels as sharp peaks in the photonic density of states^34^. These papers provided a solid proof of the implementation of gauge fields for photons in different wavelength ranges and physical systems.

Yet in another paper, the concept has been extended to non-Abelian gauge fields by Marin Soljačić, Yi Yang and colleagues, who introduced and theoretically studied two non-Abelian generalizations of the Harper–Hofstadter model^35^. The Harper–Hofstadter model describes electrons on a 2D square lattice under a perpendicular magnetic field, which exhibits integer QHE. The magnetic field is represented as an Abelian gauge field introduced through Peierls substitution. The model is famous for, among other things, its spectrum called the Hofstadter butterfly. The authors, inspired by the real-space building blocks of non-Abelian gauge fields in a recent experiment from the same group, theoretically studied two generalizations of the model—each describing two pairs of the Hofstadter butterflies that are spin-orbit coupled, and derived the non-Abelian condition for the two models from the commutativity of their arbitrary loop operators. At zero energy, the models are gapless and host the Weyl and Dirac points protected by internal and crystalline symmetries, but at other fillings, the gapped phases of the models give rise to Z2 topological insulators.

Much of the current studies about photonic topological insulators are based on ordered bulk lattices surrounded by edges, but unconventional lattice structures such as fractal lattices and disordered lattices can give rise to distinct phenomena. A typical example is the topological Anderson insulators, as have been demonstrated in experiments already. Along this line, a few papers have been collected in this Special Issue. In ref. ^36^, Mordechai Segev and colleagues found theoretically that photonic topological insulators can also exist in fractal lattices. Fractals are geometric objects whose dimension is not an integer, with the most known example being the Cantor set. A fractal with dimension between one and two is a geometric object that does not contain an element with a well-defined area (because then its dimension would be two). This means that a photonic system with dimension between one and two has no bulk. As such, this work showing the existence of a fractal photonic topological insulator challenges the bulk-edge correspondence encountered in conventional 2D photonic topological insulators. In ref. ^37^, Yidong Chong, Baile Zhang, and colleagues investigated how disorder impacts the formation of topological edge states. The understanding of topological insulators (including their photonic realizations) relies typically on the topological band theory, which does not apply to amorphous phases of matter such as those formed in non-crystalline lattices without long-range order. However, by gradually deforming the amorphous lattice through a glass-to-liquid transition, the authors observed the closing of the mobility gap and the disappearance of the topological edge states. Such topological states persisted into the amorphous regime before the transition, which illustrated the key role of short-range order in the formation and destruction of topological edge states. In ref. ^38^, Mikael Rechtsman, Alexander Cerjan, and co-authors explored the bulk Thouless pumping (a scheme to realize topological transport in 1D settings) in the presence of disorder in arrays of evanescently-coupled optical waveguides. The authors found that pumping is quite robust to the increase of the degree of disorder. When disorder is sufficiently strong to reduce the bulk mobility gap on the scale of the modulation frequency of the system, the pumping becomes strongly affected by disorder. In the experiment, the authors realized a near-full-unit-cell transport per pump cycle for a physically relevant class of initial conditions, suggesting that the scheme could be used for designing novel disorder-resistant slow light devices.

Despite of the rapid development in topological photonics, much of the venture in this area has taken place mainly in the linear-optics regime. Recently, researchers have started to explore the interplay between topology and nonlinearity, focusing on nonlinear topological photonics^12^. As a typical example, in ref. ^39^, Daohong Song, Hrvoje Buljan, Zhigang Chen, and colleagues developed a lattice laser-writing technique with a weak continuous-wave laser and demonstrated nontrivial coupling of light by nonlinearity into a topological interface defect in a photonic Su-Schrieffer-Heeger lattice, which is otherwise not accessible due to topological protection. The authors further developed a general theoretical framework for interpreting the nonlinear mode-coupling dynamics and introduced the concepts of inherited and emergent topological phenomena, which is applicable to other nonlinear topological systems beyond photonics.

Perhaps, one of the most epoch-making developments in the broader field of topological/non-Hermitian photonics comes with the realizations of topological insulator lasers^15–24^. Compared with the conventional laser systems, topological lasers exhibit superior features such as reduced lasing threshold, robust performance, high efficiency, and single-mode operation. In ref. ^40^, Yuri Kivshar, Hong-Gyu Park and colleagues proposed and demonstrated room-temperature lasing from active nanophotonic topological cavities based on III–V semiconductor quantum wells and two interfaced valley-Hall photonic structures. The authors showed a narrow spectrum, high coherence, and low-threshold lasing under uniform pumping from high-quality cavity modes hosted within the topological bandgap of the structure. The development in topological lasers could lead to a step further toward topologically controlled ultrasmall light sources with nontrivial radiation characteristics, promising for integrated nanophotonics.

Another example of prospective applications of topological photonics is based on the concept of photonic crystal fibers. In ref. ^41^, Ling Lu and colleagues proposed a judiciously designed topological bandgap fiber with its bandgap in a different direction opened by the generalized Kekule modulation on a Dirac lattice with a different phase. The authors showed that the existence of mid-gap defect modes is guaranteed to guide light at the core of this kind of Dirac-vortex fiber, with the number of guiding modes equal to the winding number of the spatial vortex. Such topological fiber can be readily implemented with silica capillaries and could bring about new solutions to single-polarization single-mode design in microstructured fibers.

In the areas “beyond” topological photonics, there were many presentations covering a wide range of interesting topics at the two workshops. A highlight of the Tianjin workshop was the plenary talk by Michael Berry on “Geometric phases and the separation of the world”, and at the Daejeon workshop was the colloquium talk by Franco Nori on “Quantum simulation using superconducting electronic circuits”. Collected in this Special Issue are two papers from other plenary talks at the Tianjin workshop: in ref. ^42^, Vladimir Shalaev, Alexandra Boltasseva, and colleagues developed a plasmonic-enhanced graphene photodetector with a 25-fold increase in photocurrent generation compared to conventional graphene devices, and the proposed device concept may lead to promising applications toward compact, ultra-broadband and ultrafast photodetectors; in ref. ^43^, Nader Engheta and Victor Pacheco-Peña developed an approach called “temporal aiming”, in which the electromagnetic wavepackets can be steered and redirected by temporally changing the relative permittivity of metamaterials between isotropic to anisotropic values, and the proposed ideas may find applications in integrated photonics where ushering and guiding wavepackets at will is much desired.

This special issue collection includes mainly some selected work reported at the two international workshops, highlighting a few new trends in topological photonics. Clearly, it is by no means an attempt to include all the latest advances in this field. We appreciate very much the great efforts and contributions of all authors, and hope that this special issue will provide a good reference as well as fuel the inspiration for researchers in this hot and dynamically changing field of topological photonics and beyond.
